# The complete chloroplast genome sequence of an endemic monotypic genus *Hagenia* (Rosaceae): structural comparative analysis, gene content and microsatellite detection

**DOI:** 10.7717/peerj.2846

**Published:** 2017-01-10

**Authors:** Andrew W. Gichira, Zhizhong Li, Josphat K. Saina, Zhicheng Long, Guangwan Hu, Robert W. Gituru, Qingfeng Wang, Jinming Chen

**Affiliations:** 1 Key Laboratory of Aquatic Botany and Watershed Ecology, Wuhan Botanical Garden, Chinese Academy of Sciences, Wuhan, China; 2 University of Chinese Academy of Sciences, Beijing, China; 3 Sino-Africa Joint Research Center, Chinese Academy of Sciences, Wuhan, China; 4 Department of Botany, Jomo Kenyatta University of Agriculture and Technology, Nairobi, Kenya

**Keywords:** Chloroplast genome, *Hagenia abyssinica*, Afromontane, Rosaceae, Phylogeny, East Africa

## Abstract

*Hagenia* is an endangered monotypic genus endemic to the topical mountains of Africa. The only species, *Hagenia abyssinica* (Bruce) J.F. Gmel, is an important medicinal plant producing bioactive compounds that have been traditionally used by African communities as a remedy for gastrointestinal ailments in both humans and animals. Complete chloroplast genomes have been applied in resolving phylogenetic relationships within plant families. We employed high-throughput sequencing technologies to determine the complete chloroplast genome sequence of *H. abyssinica.* The genome is a circular molecule of 154,961 base pairs (bp), with a pair of Inverted Repeats (IR) 25,971 bp each, separated by two single copies; a large (LSC, 84,320 bp) and a small single copy (SSC, 18,696). *H. abyssinica*’s chloroplast genome has a 37.1% GC content and encodes 112 unique genes, 78 of which code for proteins, 30 are tRNA genes and four are rRNA genes. A comparative analysis with twenty other species, sequenced to-date from the family Rosaceae, revealed similarities in structural organization, gene content and arrangement. The observed size differences are attributed to the contraction/expansion of the inverted repeats. The translational initiation factor gene (*infA*) which had been previously reported in other chloroplast genomes was conspicuously missing in *H. abyssinica*. A total of 172 microsatellites and 49 large repeat sequences were detected in the chloroplast genome. A Maximum Likelihood analyses of 71 protein-coding genes placed *Hagenia* in Rosoideae. The availability of a complete chloroplast genome, the first in the Sanguisorbeae tribe, is beneficial for further molecular studies on taxonomic and phylogenomic resolution within the Rosaceae family.

## Introduction

*Hagenia* is a monotypic genus under the Rosaceae family which is one of the largest and most economically important families with over 100 genera and more than 3,100 species (Potter et al., 2007). Many genera in this family include species that have been domesticated for fruit production, medicinal values and for ornamental purposes. *Hagenia abyssinica* (Bruce) J.F. Gmel. is a dioecious tree species, endemic to the isolated Afromontane forests of Africa at elevations between 2,300 and 3,400 m above sea level (Hedberg, 1969). The species is characterized by large reddish female and whitish male inflorescences, and its pollen and seeds are dispersed by wind (Negash, 1995). *H. abyssinica* has traditionally been used by the African communities as a source of herbal medicine for the treatment of gastrointestinal ailments in both man and animals (Assefa, Glatzel & Buchmann, 2010; Nibret & Wink, 2010; Scantlebury et al., 2013; Feyssa et al., 2015). Over the past few decades, there has been a vast reduction of natural populations of this species resulting from overharvesting, selective logging and habitat destruction. Consequently, *H. abyssinica* is now listed in the Red List of endangered species in Ethiopia and other regions where assessment has been done in eastern Africa (Negash, 1995; Vivero, Kelbessa & Demissew, 2005; Seburanga, Nsanzurwimo & Folega, 2014).

Several studies employing both traditional (morphology and chromosome number) and molecular techniques have been conducted aiming to assess the relationships within the family Rosaceae (Rosales). Molecular studies have analysed both the nuclear and plastid DNA. One of the early molecular phylogenetic studies in Rosaceae used chloroplast sequences of a single gene- *rbcL*- to assess the traditional subfamilial classification and to shed light on some problematic taxa within this family (Morgan, Soltis & Robertson, 1994). Further molecular phylogenetic analyses have been conducted in Rosaceae utilizing various coding and non-coding sequences, from the nuclear and/or the chloroplast genomes (Evans, 1999; Evans et al., 2000; Potter et al., 2002). In these studies, some of the traditional groupings were validated e.g., sub-dividing the family into Rosoideae, Maloideae, Spiraeoideae and Amygdaloideae by Schulze-Menz (1964). However, major contraditions between traditional and molecular-based studies were noted and significant differences were also observed between the molecular studies probably due to the use of different but limited number of partial DNA sequences. Additional clarifications in the phylogeny and classification of Rosaceae were made in Potter et al. (2007), where three sub-families (Rosoideae, Dryadoideae and Spiraeoideae) were supported. These studies have greatly boosted our understanding of phylogenetic relationships in Rosaceae. However, certain clades, as discussed in Potter et al. (2007), remain ambiguously classified while others are weakly supported.

The first complete sequences of cpDNA were reported three decades ago in *Marchantia polymorpha* (Ohyama et al., 1986) and in *Nicotiana tabacum* (Shinozaki et al., 1986), and since then there had been gradual increase in the number of sequenced complete chloroplast genomes. However, the advent of next-generation DNA sequencing technologies significantly reduced the cost and time involved in DNA sequencing (Shendure & Ji, 2008; Daniell et al., 2016. Consequently, the number of species with complete sequenced nuclear and organellar genomes has rapidly increased. The chloroplast genome is circular and it is characterized by a quadripartite structure with two inverted repeats (IRa and IRb) that are separated by one Large Single Copy region (LSC) and one Small Single Copy region (SSC). The size of complete chloroplast genome sequences range between 107 and 217 kb. Genome size fluctuations could be attributed to; duplication of genes and occurrence of small repeats (Xu et al., 2015), gene loss and/or transfer to other genomes (Stegemann et al., 2003) and the contraction/expansion of the inverted repeats at the four IR/SC junctions (Downie & Jansen, 2015).

In angiosperms, one of the key traits of the organellar DNA is uniparental inheritance; thus, it is well conserved and allows for the development of informative universal markers. These attributes make the chloroplast genome more valuable for application in various molecular studies in plants e.g., DNA barcoding, outlining species evolutionary histories, molecular phylogenetics and population genetics. Recently, complete chloroplast genomes have extensively been used in plant identification and resolution of phylogenetic relatioships at different taxonomic levels (Jansen et al., 2007; Yang et al., 2013; Zhang et al., 2016).

Currently, whole chloroplast genomes of several species from the Rosaceae family representing nine genera have been studied and deposited at the GenBank database (NCBI; http://www.ncbi.nlm.nih.gov/). However, only a few of these species, such as *Fragaria chiloensis* (Salamone et al., 2013) and *Potentilla micrantha* (Ferrarini et al., 2013), are from the sub-family Rosoideae whose whole cpDNA have been sequenced. At present, none from the Agrimoniinae clade has been sequenced and the closest studied genus- to *Hagenia*- is* Rosa* (Yang, Li & Li, 2014). Therefore, the objectives of this study were to establish and characterize the organization of the complete chloroplast genome sequence of *H. abyssinica* and to compare its structure, gene arrangement and IR boarders to other members of the Rosaceae family. Because this is the first whole chloroplast genome presented from the Sanguisorbeae tribe, it will act as a reference chloroplast genome within the tribe.

## Materials and Methods

### DNA extraction and sequencing

Young leaf samples were collected from natural populations of *Hagenia abyssinica* in Mt. Kenya (Kenya; 00°09′35.29″S/037°26′56.40″E). A voucher specimen (SAJIT_001956) was deposited at the Herbaria of Wuhan Botanical Garden, Chinese Academy of Sciences (HIB). Total genomic DNA was extracted from 100–150 mg of leaves using the MagicMag Genomic DNA Micro Kit (Sangon Biotech Co., Shanghai, China) following the manufacturer’s instructions. The quality of the extracted DNA was checked by gel electrophoresis and confirmed using Qubit DNA Assay kit in Qubit 2.0 Fluorometer (Life Technologies, San Diego, CA, USA). Paired-end library was constructed using an Illumina TruSeq Library preparation kit (Illumina, San Diego, CA, USA) following the manufacturer’s protocol. Genomic DNA was sequenced using the Illumina Hiseq 2500 platform (Illimina Inc.), yielding 41.2 million 150-bp paired-end reads from a library of ∼350  bp DNA  fragment.

### Genome assembly and annotation

We used a reference-guided strategy to assemble the chloroplast genome. Firstly, whole clean data were identified using BLAST (http://blast.ncbi.nlm.nih.gov/) with default parameters, by searching against the plastome sequences of *Fragaria chiloensis* (JN884816). The generated contigs were sorted, and the chloroplast genome reads were extracted by mapping the contigs against already available chloroplast sequences of *Fragaria chiloensis* (JN884816; Salamone et al., 2013) using the Basic Local Alignment Search Tool (BLAST; http://blast.ncbi.nlm.nih.gov/) with default parameters. The retained high quality reads were then assembled into non- redundant contigs using Velvet 1.2.10 (Zerbino & Birney, 2008) with K-mer length of 95–107. Five contigs whose size ranged between 1,960 and 47,845 bp were then blasted against *Fragaria chiloensis* and *Pyrus pyrifolia* (AP012207; Terakami et al., 2012). Specific primers were designed using PRIMER 5.0 (PREMIER Biosoft International, CA, USA) and used in Polymerase Chain Reaction to fill gaps between the contigs and to validate the joints between the IR/LSC and IR/SSC, based on the Sanger sequencing technique. The primer sequences used in filling the gaps and validating the IR/SC junctions are listed in File S1.

The assembled chloroplast genome was annotated using an online-based program: the Dual OrganellarGenomMe Annotator (DOGMA; http://dogma.ccbb.utexas.edu/, Wyman, Jansen & Boore, 2004) followed by manual corrections of the start, stop codons and the boundaries between the introns and exons based on homologous genes from other sequenced chloroplast genomes. Protein coding, transfer RNA (tRNA) and ribosomal RNA (rRNA) genes were also predicted in DOGMA with default parameters. The tRNA genes were further verified using tRNAscan-SE 1.23 program (http://lowelab.ucsc.edu/tRNAscan-SE/; Schattner, Brooks & Lowe, 2005). Finally, a circular gene map was constructed using the OrganellarGenomeDRAW software (OGDRAW; http://ogdraw.mpimp-golm.mpg.de). The complete chloroplast genome sequence of *H. abyssinica* can be found in GenBank under the accession number KX008604.

### Microsatellite discovery and comparative analyses

The Perl script based Microsatellite identification tool (MiSa) (Thiel et al., 2003) was used to detect microsatellites with minimal iterations of eight repeat motifs for mononucleotides, five for dinucleotides, four for trinucleotides and three for Tetra-, Penta- and hexa-nucleotides. The location and size of the repeating sequences (forward, reverse, complementary and palindromic) were visualized in REputer (Kurtz & Schleiermacher, 1999) with minimal repeat size set at ≥15 and Hamming distance at 3.

To highlight structural differences and similarities between *H. abyssinica* and other already sequenced chloroplast genomes in Rosaceae family, we retrieved 20 currently available complete chloroplast genomes from the NCBI (Table 1) and conducted comparative analyses. Special attention was paid to the sizes of the entire complete genomes and inverted repeats, the location of the IR/SC junctions and arrangement of genes adjacent the IR/SC boarders.

**Table 1:** Comparison of complete chloroplast genomes in 21 taxa of Rosaceae; size, contraction/expansion of the inverted repeats and gene arrangement around the four IR/SC junctions.

							IRa/LSC		IRa/SSC		IRb/SSC	IRb/LSC		
GenBank No.	Species	Genome size	LSC length	SSC Length	IR length	Sub-family	*rps19 (bp)*	*rpl2 (bp)*	*Ψycf1 (bp)*	*ndhF (bp)*	*ycf1 (bp)*	*rpl2 (bp)*	Ψ*rps19 (bp)*	*trnH-GUG (bp)*
KU851961	*Malus prunifolia*	160,041	88,119	19,204	26,359	**Spiraeoideae**	119		9	11	1,073	−190	129	−38
AP012207	*Pyrus pyrifolia*	159,922	87,901	19,237	26,392		21	−92	−90	110	975	−289	149	−3
HG737342	*Pyrus spinosa*	159,161	87,694	19,205	26,396		8	−79	−114	113	493	−520	141	−91
KC571835	*Prinsepia utilis*	159,328	85,239	18,485	26,302		178	−107	−110	−32	978	−3,398	179	−91
KP760072	*Prunus padus*	158,955	87,667	18,872	26,208		38	−109	5	19	1,035	−109	*	−22
KP760073	*Prunus serrulata* var. *spontanea*	157,882	85,969	19,121	26,396		177	−248	13	−2	1,045	−248	162	−24
KP760070	*Prunus yedoensis*	157,859	85,978	19,121	26,380		179	−250	18	−21	1,040	−250	185	−46
KP760071	*Prunus maximowiczii*	157,852	85,848	19,134	26,435		216	−287	13	−2	1,045	−287	221	−21
KP760075	*Prunus surbhirtela*	157,833	85,952	19,121	26,381		179	−250	17	−21	1,040	−250	185	−46
HQ336405	*Prunus persica*	157,790	85,968	19,060	26,381		95	−167	−81	96	946	−338	182	−3
KF990036	*Prunus kansuensis*	157,736	85,755	19,209	26,386		181	−252	5	9	1,050	−338	182	−79
KF765450	*Prunus mume*	157,712	85,830	19,094	26,394		196	−267	−102	−17	1,018	−298	206	−2
KF753637	*Rosa odorata* var. *gigantea*	156,634	85,767	18,761	26,053	**Rosoideae**	−14	−55	57	−44	1,105	−54	*	−4
JQ041763	*Pentactina rupicola*	156,612	84,970	18,942	26,350		152	−223	0	40	1,057	−222	151	−35
JF345175	*Fragaria vesca* var. * vesca*	155,691	85,606	18,175	25,555		−10	−55	31	−93	1,091	−54	*	−35
JN884817	*Fragaria virginiana*	155,621	85,587	18,146	25,944		−13	−54	12	−33	1,091	−54	*	−34
JN884816	*Fragaria chiloensis*	155,603	85,568	18,147	25,944		−13	−54	12	−33	1,091	−54	*	−34
KC507760	*Fragaria mandshurica*	155,596	85,515	18,171	25,955		−13	−54	12	59	1,091	−54	*	−34
KC507759	*Fragaria iinumae*	155,554	85,569	18,059	25,963		−13	−55	21	−50	1,091	−54	*	−34
KX008604	*Hagenia abyssinica*	154,961	84,320	18,696	25,971		−130	−57	53	12	1,082	−57	*	−3
HG931056	*Potentilla micrantha*	154,959	85,137	18,762	25,530		−1,016	−489	−476	400	1,040	−60	*	−3

**Notes.**

SSC small single copy LSC large single copy IR inverted repeat (a/b) bp base pairs Ψ pseudogene; * missing]

The negative (−) numbers indicate the size of the gap between the IR/SC junction and the gene involved. Except for Ψ*rps19*, the other numbers shows the size of the gene that is located in the IR.

To gain insight into the relationship of members of Rosaceae, a Maximum Likelihood (ML) phylogenetic tree was reconstructed. We used 71 protein-coding genes common in all the 21 species of Rosaceae. Two taxa; *Morus indica* (Moraceae) and *Eleagnus macrophylla* (Eleagnaceae), from the clade Rosales, were used as outgroups. All the PCGs were aligned in MUSCLE (Edgar, 2004) with default settings and appropriately edited manually. The jModelTest 2.1.7 program (Darriba et al., 2012) was used to select the best fitting substitution model based on the Akaike information criterion (Posada & Buckley, 2004). The best-fitting substitution model GTR + I + G model of all genes was used. The GTR + I + G model was used for ML analyses implemented in RAxML 8.0.20 following instructions from the manual (Stamatakis, 2014). A bootstrap analysis was performed with 1,000 replications.

## Results and Discussion

### Genome content and organization

**Figure 1:**
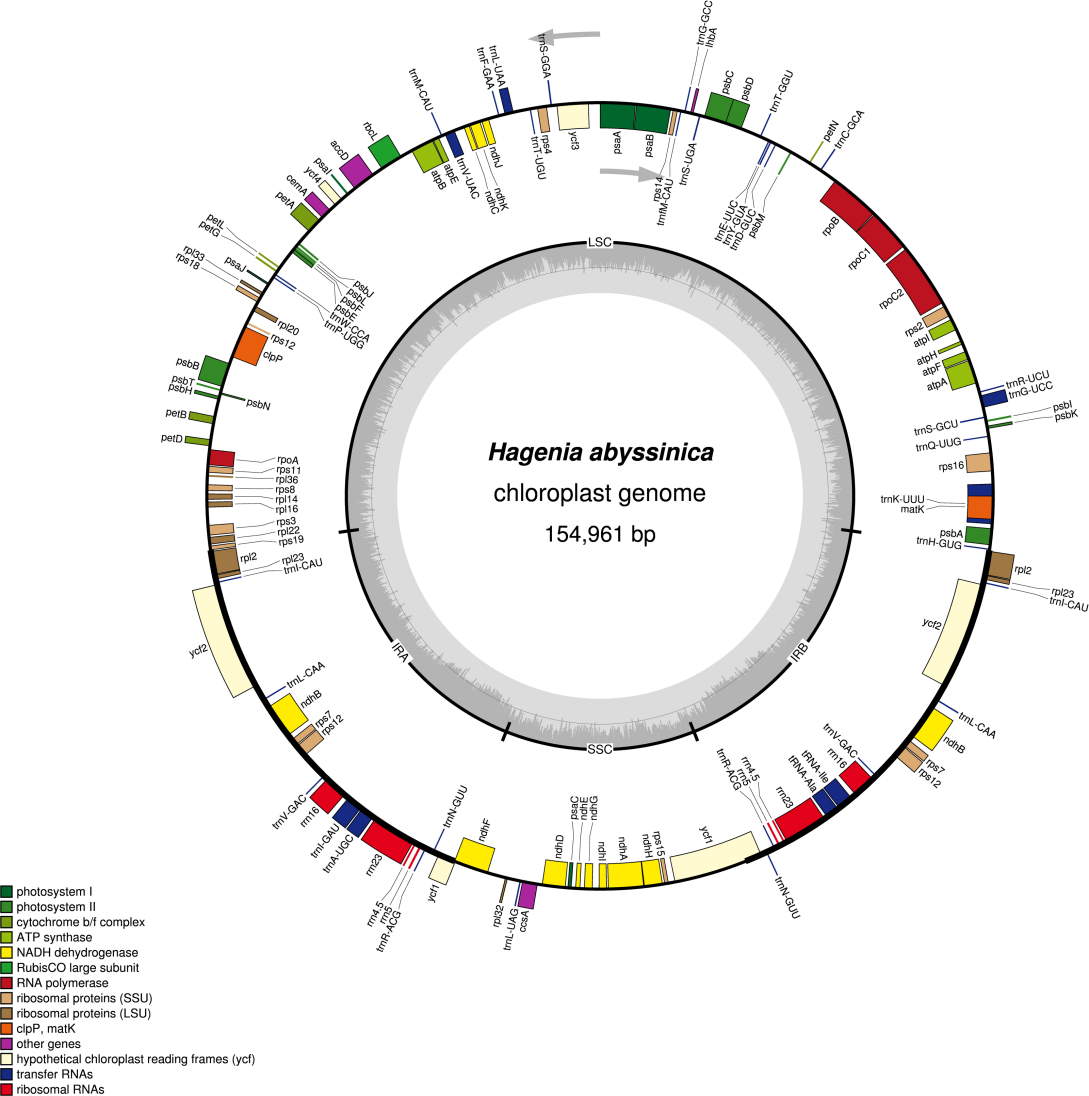
A gene map of *Hagenia abyssinica* chloroplast genome. The GC content is represented by the dark shading on the inner side of the small circle, whereas the light shading represents the AT content. The genes are color-coded based on different functional group.

The complete chloroplast genome of *H. abyssinica* exhibited a double- stranded circular DNA molecule, with a total length of 154,961 bp (Fig. 1). It also displayed a quadripartite structure, typical to chloroplast genomes of most terrestrial plants. The chloroplast genome possesses a pair of inverted repeats (IRa and IRb) of 25,971 bp each. The IRs are separated by a large single copy (LSC) and a small single copy (SSC) with 84,320 bp and 18,696 bp respectively (Fig. 1). The total GC content for this chloroplast genome is 37.1%, which is consistent with those from other species in Rosaceae. The chloroplast genome of *H. abyssinica* encodes 129 genes (excluding the *ORFs* and the the hypothetical genes; *ycf68* and *ycf15*), comprising 78 unique protein—coding genes (PCGs), 30 unique tRNA and 4 rRNA genes (Table 2). In total there were 17 duplicated genes, 7 of which code for protein in the IRs including *rpl2, rpl23, ycf2, ndhB, rps7, rps12,* and *ycf1*, 6 tRNA and 4 rRNA were also among the duplicates in the IRs. The gene order in the SSC region begins with *ndhF,* followed by *rpl32, trnL, ccsA, ndhD, PsaC, ndhE, ndhG, ndhI, ndhA, ndhH and rps15* and ends with *ycf1*. Six protein coding genes contained either one intron (*rps16, rpl2, rpl23, rpoC1, ndhA* and *ndhB*) or two introns (*clpP*). The hypothetical gene * ycf3*, contained two introns (Table 2). The *rps12* gene is trans-spliced with the 3′ exon being duplicated in the IR, while the 5′ end is located at the LSC region.

**Table 2:** List of genes in the chloroplast genome of *Hagenia abyssinica*.

Category	Gene type	Gene						
Self-replication	Ribosomal RNA	*rrn16*	*rrn23*	*rrn4.5*	*rrn5*			
	Transfer RNA	trnA-UGC^*^	*trnfM-CAU*	trnI-GAU^*^	*trnM-CAU*	*trnR-ACG*	*trnS-UGA*	
		*trnC-GCA*	trnG-GCC^*^	trnK-UUU^*^	*trnN-GUU*	*trnW-CCA*	*trnT-GGU*	
		*trnD-GUC*	*trnG-UCC*	*trnL-CAA*	*trnY-GUA*	*trnR-UCU*	*trnT-UGU*	
		*trnE-UUC*	*trnH-GUG*	trnL-UAA^*^	*trnP-UGG*	*trnS-GCU*	*trnV-GAC*	
		*trnF-GAA*	*trnI-CAU*	*trnL-UAG*	*trnQ-UUG*	*trnS-GGA*	trnV-UAC^*^	
	Small ribosomal units	*rps11*	*rps12*		*rps14*	*rps15*	rps16^*^	*rps18*
		*rps19*	*rps2*	*rps3*	*rps4*	*rps7*	*rps8*	
	Large ribosomal units	*rpl14*	*rpl16*	rpl2^*^	*rpl20*	*rpl22*	*rpl23*	*rpl32*
		*rpl33*	*rpl36*					
	RNA polymerase sub-units	*rpoA*	*rpoB*	rpoC1^*^	*rpoC2*			
Photosynthesis genes	NADH dehydrogenase	ndhA^*^	NdhB^*^	*ndhC*	*ndhD*	*ndhE*	*ndhF*	
		*ndhG*	*ndhH*	*ndhI*	*ndhJ*	*ndhK*		
	Photosystem I	*psaA*	*psaB*	*psaC*	*psaI*	*psaJ*	ycf3^**^	*ycf4*
	Photosystem II	*psbA*	*psbB*	*psbC*	*psbD*	*psbE*	*psbF*	*psbH*
		*psbI*	*psbJ*	*psbK*	*psbL*	*psbM*	*psbN*	*psbT*
		*lbhA*						
	Cytochrome b/f complex	*petA*	*petB*	*petD*	*petG*	*petL*	*petN*	
	ATP synthase	*atpA*	*atpB*	*atpE*	*atpF*	*atpH*	*atpI*	
	Large subunit of rubisco	*rbcL*						
Other genes	Maturase	*matK*						
	Protease	clpP^**^						
	Acetyl-CoA-carboxylase sub-unit	*accD*						
	Envelope membrane protein	*cemA*						
	Component of TIC complex	*ycf1*						
	c-type cytochrome synthesis	*ccsA*						
Unknown	hypothetical genes reading frames	*ycf2*						

**Notes.**

* Genes with a single intron.

** Genes with two introns.

### Discovery of SSRs

Microsatellite markers are considered ideal for plant molecular studies due to their high mutation rates, multi- allelism and locus- specificity (Varshney, Graner & Sorrells, 2005; Govindaraj, Vetriventhan & Srinivasan, 2015) and thus highly informative. Recently, seventeen species-specific nuclear SSR markers have been reported for this species (Gichira et al., 2016). In a previous study, three concensus chroloplast microsatellite markers had been used to study genetic diversity of *H. abyssinica* (Ayele et al., 2009). Chloroplast-derived microsatellite markers have generated great impact on population genetics, plant evolutionary studies and phylogenetics (Provan, Powell & Hollingsworth, 2001). In this study, a total of 172 SSR repeat motifs were discovered (Table 3).

**Table 3:** Characterization of simple sequence repeats discovered in the chloroplast genome of *Hagenia abyssinica*.

Microsatellite sequences	Number of repeats													Total
	**3**	**4**	**5**	**6**	**7**	**8**	**9**	**10**	**11**	**12**	**13**	**14**	**15**	
A	–	–	–	–	–	25	14	10	5	6	3	1	1	65
C	–	–	–	–	–	6	3	1	–	–	–	–	–	10
G	–	–	–	–	–	3	1	–	–	–	–	–	–	4
T	–	–	–	–	–	30	20	14	6	2	1	1	1	75
AT	–	–	2	2	–	–	–	–	–	–	–	–	–	4
TA	–	–	5	–	–	–	–	–	–	–	–	–	–	5
TC	–	–	1	–	–	–	–	–	–	–	–	–	–	1
AAAT	2	–	–	–	–	–	–	–	–	–	–	–	–	2
AATA	1	–	–	–	–	–	–	–	–	–	–	–	–	1
ATGT	1	–	–	–	–	–	–	–	–	–	–	–	–	1
TAAA	1	–	–	–	–	–	–	–	–	–	–	–	–	1
TAAT	1	–	–	–	–	–	–	–	–	–	–	–	–	1
TTTA	2	–	–	–	–	–	–	–	–	–	–	–	–	2
Total														172

Mononucleotides had the highest number of repeats (88%), most of which had the A/T repeat type which is in line with the findings of a previous study that polyA and polyT repeats dominate in chloroplast microsatellites (Cai et al., 2008). A total of 5.8% represented dinucleotides while the rest were tetranucleotides, nine of the dinucleotides had the AT/TA repeat motif while AAAT/TTTA motifs dominated among the tetranucleotides. There were no trinucleotide repeats detected in *H. abyssinica*’s chloroplast genome. Repeat motifs are potential molecular tools for studying recombination and rearrangement in genomes (Smith, 2002). In addition to SSRs, a total of 49 repeat sequences with at least 21 bp were identified by REPuter. The repeat units had a sequence identity of ≥90% and their sizes ranged from 21 to 69 bp. The 49 repeats constituted 22 palindrome (inverted) repeats, 19 direct (forward) repeats, seven reverse repeats and one complementary repeat (Table 4). The majority of the identified repeats were located in the non-coding regions of the genome which is in line with observations made in other chloroplast genomes of angiosperms (Provan, Powell & Hollingsworth, 2001; George et al., 2015). This trend of cpSSR distribution, has been observed in other chloroplast genomes species in Rosaceae suggesting that they may be suitable for conducting population genetic diversity, phylogenetic and evolutionary studies in species under this family.

**Table 4:** List and location of long repeat sequences in the chloroplast genome of *Hagenia abyssinica*.

Repeat size (bp)	Repeat 1 start	Repeat 2 start	Repeat type	Location 1	Location 2
69	26,722	26,745	F	IGS (*rpoB-trnC-GCA*)	IGS (*rpoB-trnC-GCA*)
67	52,510	52,510	P	IGS (*trnM-CAU-atpE*)	IGS *(trnM-CAU-atpE*)
59	52,514	52,514	P	IGS (*trnM-CAU-atpE*)	IGS (*trnM-CAU-atpE*)
56	10,134	10,134	P	IGS (*trnR-UCU-atpA)*	IGS (*trnR-UCU-atpA*)
46	26,722	26,768	F	IGS (*rpoB-trnC-GCA*)	IGS (*rpoB-trnC-GCA*)
40	98,746	12,0719	F	IGS (*rps7-trnV-GAC*)	IGS (*ndhA-ndhA*)
40	12,0719	14,0493	P	IGS (*ndhA-ndhA*)	IGS (*trnV-GAC-rps7*)
39	44,079	98,748	F	*ycf3*	IGS (*rps7-trnV-GAC*)
39	44,079	14,0492	P	*ycf3*	IGS (*trnV-GAC-rps7*)
38	44,079	12,0721	F	*ycf3*	IGS *(ndhA-ndhA*)
37	12,859	12,859	P	IGS (*atpF-atpH*)	IGS (*atpF-atpH*)
34	8342	45,240	P	IGS (*psbI-trnS-GCU*)	*trnS-GGA*
30	8,346	45,240	P	IGS (*psbI-trnS-GCU*)	*trnS-GGA*
30	10,7688	10,7720	F	IGS (*rrn4.5-rrn5*)	IGS (*rrn4.5-rrn5*)
30	10,7688	13,1529	P	IGS (*rrn4.5-rrn5*)	IGS (*rrn5-rrn4.5*)
30	10,7720	13,1561	P	IGS (*rrn4.5-rrn5*)	IGS (*rrn5-rrn4.5*)
30	13,1529	13,1561	F	IGS (*rrn5-rrn4.5*)	IGS (*rrn5-rrn4.5*)
29	35,992	36,014	F	IGS (*trnS-UGA-lbhA*)	IGS (*trnS-UGA-lbhA*)
28	67,251	67,275	F	IGS (*psaJ-rpl33*)	IGS (*psaJ-rpl33*)
30	47,381	47,381	P	IGS (*trnT-UGU-trnL-UAA*)	IGS (*trnT-UGU-trnL-UAA*)
24	36,841	36,841	P	IGS (*trnG-UCC-trnfM-CAU*	IGS (*trnG-UCC-trnfM-CAU)*
24	67,255	67,279	F	IGS (psaJ-rpl33)	IGS (*psaJ-rpl33*)
27	9,748	36,800	F	IGS (*trnS-GCU-trnG-GCC*)	*trnG-UCC*
29	7,294	12,5722	R	IGS (*trnQ-UUG-psbK*)	*ycf1*
29	8,344	35,778	F	IGS (*psbI-trnS-GCU*)	*trnS-UGA*
23	26,722	26,791	F	IGS (*rpoB-trnC-GCA*)	IGS (*rpoB-trnC-GCA*)
31	96,104	96,104	P	IGS (*ndhB-ndhB*)	IGS (*ndhB-ndhB*)
31	96,104	14,3144	F	IGS (*ndhB-ndhB*)	IGS (*ndhB-ndhB*)
31	14,3144	14,3144	P	IGS (*ndhB-ndhB*)	IGS (*ndhB-ndhB*)
28	10,275	10,275	P	IGS (*trnR-UCU-atpA*)	IGS (*trnR-UCU-atpA*)
28	59,119	59,119	P	IGS (*accD-psaI*)	IGS (*accD-psaI*)
22	35,852	45,182	P	IGS (*rpoB-trnS-UGA*)	IGS (*ycf3-trnS-GGA*)
22	56,966	56,966	R	IGS (*rbcL-accD*)	IGS (*rbcL-accD*)
22	80,656	80,656	P	IGS (*rps8-rpl14*)	IGS (*rps8-rpl14*)
25	8,348	35,782	F	*trnS-GCU*	*trnS-UGA*
25	35,782	45,243	P	*trnS-UGA*	*trnS-GGA*
30	7,017	7,021	R	IGS (*rps16-trnQ-UUG*)	IGS (*rps16-trnQ-UUG*)
30	28,761	98,934	R	IGS (*petN-psbM*)	IGS (*rps7-trnV-GAC*)
30	28,761	14,0315	C	IGS (*petN-psbM*)	IGS (*trnV-GAC-rps7*)
30	39,041	41,265	F	*psaB*	*psaA*
30	81,696	12,0708	F	IGS (*rpl16-rps3*)	IGS (*ndhA-ndhA*)
27	10,259	36,722	P	IGS (*trnR-UCU-atpA*)	IGS (*lbhA-trnG-UCC*)
27	56,961	56,966	R	IGS (*rbcL-accD*)	IGS (*rbcL-accD*)
21	8,352	35,786	F	*trnS-GCU*	*trnS-UGA*
21	12,792	68,281	F	IGS (*atpF-atpH*)	*rps18*
21	30,092	30,092	R	IGS (*psbM-trnD-GUC*)	IGS (*psbM-trnD-GUC*)
21	35,786	45,243	P	*trnS-UGA*	*trnS-GGA*
21	63,682	63,682	R	*psbJ*	*psbJ*
29	32,026	32,026	P	IGS (*trnT-GGU-psbD*)	IGS (*trnT-GGU-psbD*)

**Notes.**

F forward R reverse P palindromic C complementary

### Comparative analysis and phylogenetics

The number of species from the Rosaceae family with completely sequenced cholorplast genomes is rapidly increasing. Currently, the complete chloroplast genomes of 20 species from eight genera in two sub-families of Rosaceae family have been sequenced and deposited at GenBank (http://www.ncbi.nlm.nih.gov/). Out of the 20 species, 12 belong to the Spiraeaideae sub-family while the rest fall under the Rosoideae sub-family (Potter et al., 2007; Hummer & Janick, 2009). We compared the structure of *Hagenia*’s chloroplast genome to those available from the eight genera. The list of the species used for comparision and their accession numbers are shown in (Table 1). Characteristically, there are four junctions in the chloroplast genomes of angiosperms, due to the presence of two identical copies of the inverted repeats. However, the loss of one inverted repeat has been reported in some flowering plants e.g., in legumes (Palmer et al., 1987b). All chloroplast genomes appeared to be structurally similar with a typical quadripartite structure of two IRs separated by a LSC and a SSC. The whole genome sizes ranged from 154,959 (*Potentilla micrantha*) to 160,041 (*Malus prunifolia*) and there was a clear distinction of the sub-families based on genome sizes. Species from the Maloideae sub- family have a larger chloroplast genome compared to those from the Rosoideae. The size of *H. abyssinica’s* chloroplast genome (154,961 bp) is only 2 bp larger than that of the smallest chloroplast genome of *P. micrantha* (154,959 bp; Ferrarini et al., 2013).

Size variations of the chloroplast genome may be attributed to the expansion/contraction of the IR, with small variations (<100 bp) being common even among species under the same genus (Goulding et al., 1996). The expansion and/or contraction of the IRs is regarded as a significant evolutionary event and can be a source of polymorphic genetic markers for species identification and for analyzing phyologenetic studies in plants (Wang et al., 2008). In this study, sizes of the IRs varied from 26,435 bp in *Prunus maximowiczii* to 25,530 in *P. micrantha*. Although certain genes near the IR/SC boarders appeared to be conserved in all the species, key variations were noted in gene arrangement along the IR/SC junctions (Table 1). Two genes (*rps19* and *rpl2*) are adjacent the IRa/LSC boarder at varying positions, while the IRb/LSC junction is flanked between genes *rpl2* and *trnH-GUG* and in some cases a pseudogene (Ψ) of *rps19* gene is included in this region. This is a common feature in angiosperms, excluding monocots whose *trnH-GUG* gene is located in the IR between the genes *rpl2* and *rps19* (Goulding et al., 1996; Wang et al., 2008).

In all species from the Spiraeoideae subfamily and in one Rosoideae species—*Pentactina rupicola*—the IRa/LSC junction occurs within the coding region of the *rps19* gene resulting into the presence of Ψ*rps19* gene of various length in the IRb. This event has also been reported in the chloroplast genomes of other species e.g., *Arabidopsis thaliana* (Sato et al., 1999) and * Coffea arabica* (Samson et al., 2007). However in the other species, including *H. abyssinica* the entire *rps19* gene is located in the LSC region, leaving a gap of varying length between the 5′ end of the gene and the IRa/LSC junction, this is similar to other dicots such as *Nicotiana tabacum* (Shinozaki et al., 1986). The largest gap was 1,016 bp in *P. micrantha* followed by 130 bp in *H. abyssinica.* The *rpl2* gene is entirely located in both IRs region in all species, consequently leaving a gap of non-coding region between the IR/LSC junction and *rpl2* gene. The IRb/LSC junction is situated in the down-stream of non-coding region of the *trnH-GUG* gene in all analysed species. Those species with Ψ*rps19,* the pseudogene was located within the IR, between the *rpl2* and the *trnH-GUG.* In some dicots e.g., * Actinidia chinensis* (Yao et al., 2015), *trnH-GUG* and a section of the *psbA* occur in the inverted repeat due to expansion on the IRs into the LSC region.

In all the studied species, the IRb/SSC junction is located within the coding region of the *ycf1* gene. Consequently, the *ycf1* gene extends into the IRb at varying lengths ranging from 946 bp in *Prunus persica* to 1,091 bp in all species of genus *Fragaria*. As a result, the IRa/SSC junction is bordered by Ψ*ycf1* and gene *ndhF*, which is a general structure among the dicots e.g., tobacco and *Arabidopsis*. In *Hagenia*, the *ycf1* gene has an extension of 1,040 bp into the IRb and therefore, its Ψ*ycf1* of 1,151 bp overlaps with *ndhF* (2,234 bp) at 65 bp. The chloroplast genome of *Annona cherimola,* which is one of the largest plastid genomes with 201,723 bp, has an extremely reduced SSC (2,966 bp) due to major expansions of the IRs and most genes including the *ycf* genes have been incorporated in the IRs (Blazier et al., 2016).

Chloroplast DNA is reported to have evolved from free-living Cyanobacteria through endosymbiosis with a history of more than 1.2 billion years and since then a number of genes, initially found in the chloroplast genomes have relocated to the nuclear genome (Timmis et al., 2004), e.g., in *Arabidopsis* 18.1% of its functional nuclear genes originated from the plastid genome (Martin et al., 2002). Further studies presented more evidence on independent gene transfers from the chloroplast to the nuclear genome in rosids (Millena et al., 2001), these includes the successful transfers of *rpl22* gene in *Castanea*, *Quercus* and * Passiflora* (Jansen et al., 2011),  *infA* gene in * Arabidopsis* (Sato et al., 1999) and in Elaeagnus (Choi, Son & Park, 2015). These transfers occurred in the initial stages of plastid evolution, though a high relocation rate of non-coding DNA happens continuously (Martin et al., 2002; Timmis et al., 2004). Generally, loss and/or transfer of genes from the chloroplast genomes to the nuclear or mitochondria genomes is as a result of evolutionary events, allowing chloroplast genomes to act as valuable molecular tools in phylogenetic and evolutionary studies. Further comparative analyses revealed that the initiation factor 1 (*infA)* gene which was observed in other species of Rosaceae, is conspicuously missing from the *Hagenia* chloroplast genome. The loss/transfer of the *infA* gene, which is an essential gene in *Escherichia coli* (Cummings & Hershey, 1994), is common among the angiosperms and it is regarded as a highly mobile gene (Millena et al., 2001; Daniell et al., 2016). Therefore, besides the expansion/contraction of the IRs, gene loss provides crucial information that is essential for evolutionary studies and resolution of phylogenetic relationships among plant species.

**Figure 2:**
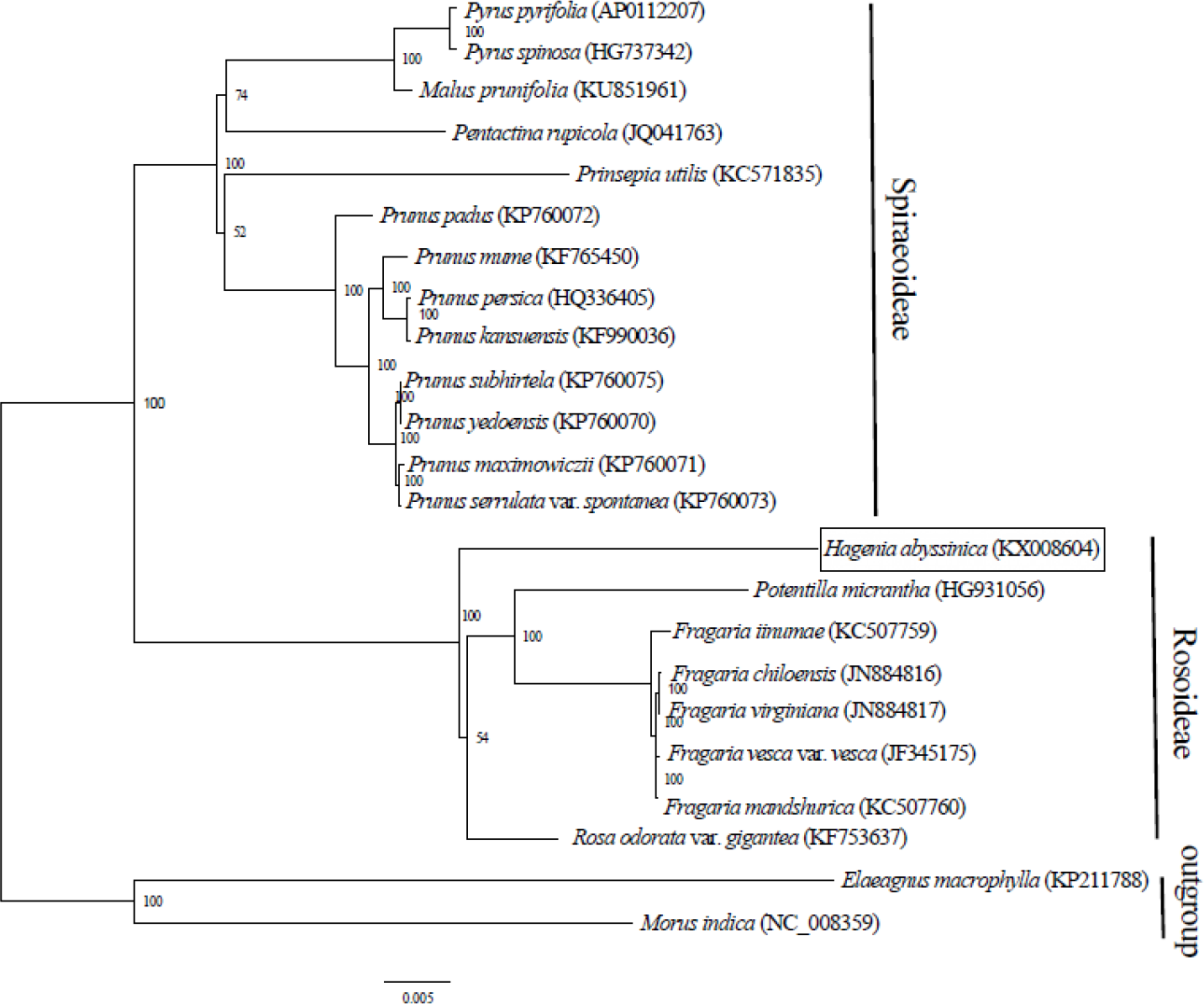
Phylogenetic relationship of 21 species of Rosaceae based on maximum likelihood analysis of 71 protein coding genes.

Complete chloroplast genome sequences provide essential genetic data for precise systematics and phylogenetic resolutions in plants. The ML phylogenetic tree that was constructed using 71 PCGs, common in all 21 taxa from Rosaceae and in two outgroups, clearly placed the Rosaceae species into two clades. The two main clades concurred with two sub-families: Spiraeoideae and Rosoideae (Fig. 2). This classification was in agreement with the phylogeny of Rosaceae (Potter et al., 2007). Previously, *Hagenia* had been classified in sub- family Rosoideae under Agrimoniinae, a subtribe in the tribe Sanguisorbeae, alongside the genera * Aremonia, Agrimonia, Leucosidea* and *Spenceria*  (Eriksson et al., 2003; Potter et al., 2007).

## Conclusion

This study provides the complete chloroplast sequences of *H. abyssinica*; an endemic species to the isolated mountains of Africa and the only species under the genus *Hagenia*. Comparative analysis revealed significant similarity in the structural organization of the chloroplast genomes in the Rosaceae family, with slight variations in size attributed to the expansion/contraction of the inverted repeats. The lost *infA* gene in the *Hagenia* chloroplast genome may have been shifted to the nuclear genome. This is the first chloroplast genome to be sequenced in the Sanguisorbeae tribe, and therefore provides valuable information for phylogenetic studies. Additionally, the data generated here provide valuable molecular markers as tools for further population genetic studies needed to support formulation of appropriate conservation measures for this endangered medicinal plant.

##  Supplemental Information

10.7717/peerj.2846/supp-1 File S1 List of primer pairs used to fill the chloroplast gaps Note: F, forward, R, reverse

## Additional Information and Declarations

### DNA Deposition

The following information was supplied regarding the deposition of DNA sequences: GenBank accession number: KX008604.

### Data Availability

The following information was supplied regarding data availability: The raw data can be found with the GenBank accession numbers in Table 1.
