# Supplementary material for: The complete chloroplast genome sequence of an endemic monotypic genus Hagenia (Rosaceae): structural comparative analysis, gene content and microsatellite detection
Source: PeerJ. 2017 Jan 10;5:e2846. doi: 10.7717/peerj.2846 (PMC5228516; doi:10.7717/peerj.2846)
Supplement: File S1 — Note: F, forward, R, reverse [file peerj-05-2846-s001.doc]

Table. List of primer pairs used to fill the chloroplast gaps

| Primer | Primer sequence (5'-3') | Annealing temp.(℃) |
| --- | --- | --- |
| Contig1/2 | F-CCTAAAGCAGTGAACCAGAT | 55 |
|  | R-ATCCCTACTTTATTGACCGC | 55 |
|  |  |  |
| Contig2/3 | F-CGAGCCAAAGTTTTAACACA | 54 |
|  | R-CAATTAACCTCTTCCGGGAT | 55 |
|  |  |  |
| Contig3/4 | F-CCAAACATTCTTCTTACGGC | 55 |
|  | R-GAGTGAAGTCGTAACAAGGT | 55 |
|  |  |  |
| Contig4/5 | F- TTGGTCCTGGTTATTCTTGG | 55 |
|  | R-ACGGCTTTCCACAGAATTAT | 55 |
|  |  |  |
| Contig5/1 | F-TATGGACAGCATCGTTGTAG | 55 |
|  | R- TTCGATATCAAACGGACCTC | 55 |

Note: F= forward, R= reverse,
